# Intrauterine growth pattern in Butajira HDSS, Southern Ethiopia: BUNMAP pregnancy cohort

**DOI:** 10.1186/s12887-023-04244-2

**Published:** 2023-08-24

**Authors:** Yalemwork G. Mengistu, Damen Hailemariam, Meselech A. Roro, Bilal S. Endris, Kokeb Tesfamariam, Seifu H. Gebreyesus

**Affiliations:** 1 Department of Public Health Nutrition and Dietetics, School of Public Health, College of Health Sciences, Addis Ababa University, Addis Ababa, Ethiopia; 2 Department of Health Systems Management and Health Policy, School of Public Health, College of Health Sciences, Addis Ababa University, Addis Ababa, Ethiopia; 3 Department of Reproductive, Family and Population Health, School of Public Health, College of Health Sciences, Addis Ababa University, Addis Ababa, Ethiopia; 4 Department of Public Health, College of Medicine and Public Health, Ambo University, Ambo, Ethiopia

**Keywords:** Intrauterine growth, Fetal growth, Growth pattern, BUNMAP, Longitudinal study, Ultrasound, Cohort study Ethiopia

## Abstract

**Background:**

Abnormal fetal growth pattern during pregnancy including excessive fetal size and intrauterine growth restrictions are the major determinants for perinatal outcomes and postnatal growth. Ultrasonography is a useful tool in monitoring fetal growth for appropriate care and interventions. However, there are few longitudinal studies using serial ultrasonography in low and middle-income countries. Moreover, the reference charts used for fetal growth monitoring in low-income countries comes from high income countries with distinct population features. Therefore, the purpose of this study was to evaluate the intrauterine growth pattern of the fetus using serial ultrasonography.

**Methods:**

We conducted a prospective community-based cohort study from March 2018 to December 2019. Pregnant women with gestational age of 24 weeks or below living in the Butajira HDSS were enrolled. We followed the pregnant women until delivery. Serial ultrasound measurements were taken, and fetal weight was estimated using the Hadlock algorithm based on biparietal diameter, head circumference, abdominal circumference, and femur length. The z-scores and percentiles of biometric measurements were calculated and compared to the INTERGROWTH-21^st^ International Standards for Fetal Growth.

**Results:**

We reviewed a total of 2055 ultrasound scans and 746 women who fulfill the inclusion criteria were involved”. We found similar distribution patterns of biometric measurements and estimated fetal weight compared to the previous study done in Ethiopia, the WHO and INTERGROWTH-21^st^ references. In our study, the 5^th^,50^th^ and 95^th^ percentiles of estimated fetal weight distribution have a similar pattern to the WHO and INTERGROWTH-21^st^ charts. The 50^th^ and 95^th^ percentile had also a similar distribution pattern with the previous study conducted in Ethiopia. We found that 10% of the fetus were small for gestational age (below the 10^th^ percentile) based on the Z-score of estimated fetal weight.

**Conclusion:**

Our study evaluated the fetal growth patterns in rural community of Ethiopia using serial ultrasound biometric measurements. We found similar IUG patterns to the WHO and INTERGROWTH-21^st^ reference standards as well as the previous study conducted in Ethiopia.

**Supplementary Information:**

The online version contains supplementary material available at 10.1186/s12887-023-04244-2.

## Introduction

Pregnancy is the time in which perinatal outcomes and postnatal growth are established [1]. Fetal size and growth trajectories are important indicators of underlying fetal health during pregnancy. Recently, fetal ultrasound has achieved a central role in diagnosis and management of fetal growth deviations. Small for gestational age (SGA) (smaller in size than the normal for their gestational age) is one of the fetal growth deviations [2]. An estimated fetal weight (EFW) below the 10^th^ centile is the most commonly used sonographic definition of small for gestational age (SGA) [3].

Detection of an abnormal growth trajectory appears to be a better evaluator of a fetus failing to achieve its growth potential. Serial observation of biometric growth patterns is the most commonly used method to determinate fetal weight [4], and to ascertain expected growth and checking that the fetus is not growth restricted [5]. Ultrasound estimation of fetal weight before birth is very widely used in today’s clinical practice, and, it is essential for the identification and management of high-risk pregnancies [6]. Ultrasound imaging could help for estimating gestational age, fetal size, and early diagnosis of fetal anomalies. This will help for the reduction of mortality rates among mothers and their babies [7].

There are very few ultrasound based multicenter global studies which were mainly conducted by WHO and the INTERGROWTH 21^st^ Project showing the intra uterine growth pattern and fetal growth chart development from few countries of the world [6, 8]. However, such global studies hide local fetal growth patterns and risk summarizing possibly dissimilar characteristics where dietary habits, adequacy of food intake, health care utilization, and cultural practices are different. Besides, in studies of the Caucasian populations, significant differences in fetal growth were found among different ethnic groups [9]. This shows that racial/ethnic-specific standards improve the precision in evaluating fetal growth.

In Ethiopia, one longitudinal study assessed intrauterine growth patterns in a rural setting and drought-affected population using serial ultrasonographic examination [10]. However, the study was only conducted on fetuses between 24 and 36 weeks of gestation and did not show the full course of growth pattern of fetus. Moreover, the growth pattern in Ethiopia and in Africa at large were not studied sufficiently and the country differences with maternal factors need to adjust the growth charts for local clinical use to increase the accuracy of fetal assessment and to avoid unnecessary obstetric interventions at the time of delivery. Therefore, this study aimed to evaluate the intrauterine growth pattern using prospective longitudinal serial ultrasound measurements in Butajira Ethiopia as the previous WHO and INTERGROWTH 21^st^ studies focused on global context and among only few countries of the world.

## Methods and materials

### Study design and setting

We conducted a prospective community-based cohort study among pregnant women in Butajira, Health and Demographic Surveillance System (HDSS) in Meskanena Mareko District of Ethiopia. Butajira is the district's capital located 135 km south of Addis Ababa, the capital city of Ethiopia. Butajira HDSS is one of the oldest surveillance sites in Africa established in 1986. The site for the HDSS has an average altitude of 2,500 m above sea level with a range of 1,500 m in the low lands to 3,500 m in the mountainous areas [11]. It consists of nine rural and one urban kebeles from different ecological zones.

This study is part of an already ongoing mother–child cohort study established in 2018 by the School of Public Health, Addis Ababa University named Butajira Nutrition, Mental health, and Pregnancy (BUNMAP) Project.

### Study population

We included pregnant women living in Butajira HDSS and followed them until delivery. Pregnant women were identified by data collectors using house to house interview of every woman of reproductive age (15–49 years) to ask if she was pregnant. If a woman responded that she was not pregnant or did not know her pregnancy status, the data collectors used a WHO checklist to determine reasonable certainty that a woman is not pregnant [12]. For those women who were suspected to be pregnant based on their interview responses or the WHO checklist, an ultrasound scan was performed to verify the pregnancy and estimate gestational age. We then included all pregnant women with a gestational age of 24 weeks or below who agreed to participate in the study.

Participant recruitment was started in March 2018, and we followed them up to December 2019. For this analysis, we included all pregnant women under the follow up of the BUNMAP project who had two or more ultrasound visits after 14 weeks of gestation. A total of 746 pregnant women with singleton fetus were included in the present analysis.

### Data collection

We collected data on baseline socioeconomic status, maternal age, maternal blood pressure, maternal hemoglobin, anthropometric measurements (maternal weight, height and mid-upper arm circumference) and obstetric history. Maternal conditions that could affect fetal growth and birth weight, such as twin pregnancy, congenital malformation, hypertensive disorders, malnutrition and anemia were assessed.

The women were assessed at nearby health facilities (health centers and health posts) They were given a follow-up card to visit the health facility to attend at least three prescheduled visits at 14, 26, and 36 weeks of gestation. They were also reminded by data collectors through home-to-home visit one day prior to their appointment date.

We collected the fetal biometric data using trans-abdominal ultrasound examination with portable Sonosite M-Turbo diagnostic imaging, and full color flow mapping ultrasound system (FUJIFILM SonoSite Inc.,Bothell, WA 98021 USA) at a time of enrolment and each subsequent visits. Ultrasound examinations were done by a midwife trained and validated by senior obstetrician. She has an experience of ultrasound scan before this study [10]. The approximate estimated date of delivery was determined by ultrasound at recruitment for the study. Intrauterine growth of the fetus was followed using serial ultrasound examination. At inclusion, gestational age (GA) and estimated date of delivery were calculated using crown-rump length (CRL) when pregnancy was between 8 and 13 weeks of gestation. After 13 weeks of gestation, fetal weight estimation was done using the Hadlock algorithms based on head circumference (HC), biparental diameter (BPD), abdominal circumference (AC), and femur length (FL) [13]. Three measurements were taken at a time for a fetus then the mean of the measurements was recorded.

Any abnormality identified during an ultra-sonographic examination was communicated to the mothers and linked to nearby hospitals for further management.

The mid-upper-arm circumference (MUAC) was determined using a standard MUAC tape.

Mid upper arm circumference** (**MUAC) of the women less than 23 cm was considered as an indicator for maternal malnutrition [14, 15]. Pregnant women with MUAC below 23 cm were linked to the nearby health institution for further nutritional management and intervention according to the national guideline of malnutrition management [16].

### Data analysis

We entered the data using Epi data software version 3.1. Then, we exported the data to the IBM SPSS Statistics for Windows, version 26 (IBM Corp., Armonk, N.Y., USA) for further cleaning and analysis. Reference curves were estimated based on centiles for each biometry and estimated fetal weight (EFW) at each gestational age from 14 to 38 weeks. Due to the small number of observations at 39 and 40 weeks of gestation, we didn’t include them in the analysis of this study. The z-scores and percentiles of biometric measurements were calculated and fitted to INTERGROWTH-21^st^ International Standards for Fetal Growth (V1.0.6257.25224) to compare the growth of many fetuses to the standards [6, 8].

Z-score of estimated fetal weight was calculated using the INTERGROWTH 21^st^ beta version 1.0 calculator for estimated fetal weight [17]. The proportion of fetuses who are small for gestational age were evaluated after identifying the 10^th^ centiles of the z-score for estimated fetal weight for gestational age 22–38 weeks. All estimated biometric measurements were computed to the nearest whole number for constructed tables of EFW and to the nearest one decimal place for BPD, HC, AC, and FL.

Pregnant women with twin pregnancies, abortion, congenital malformations and had only one ultrasound scan after 14 weeks of gestation were excluded from the analysis.

### Measurements and definitions

Small for gestational age: Small for gestational age (SGA) is defined as an estimated fetal weight below the 10^th^ percentile for gestational age [18].

Maternal under-nutrition: A pregnant mother was defined as undernourished if her mid-upper arm circumference (MUAC) fell below 23 cm [19].

Anemia: A pregnant woman was considered anemic if her hemoglobin level was < 11 g/dl [20].

## Results

**Fig. 1:**
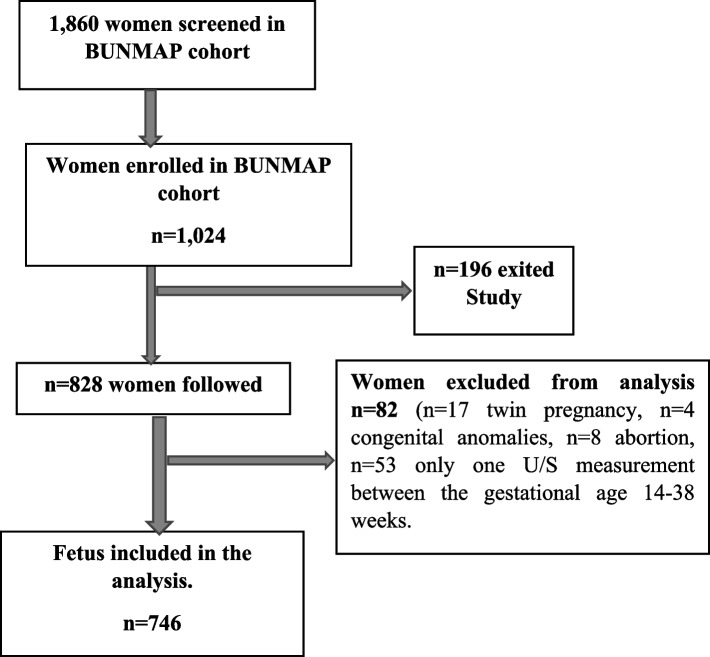
Pregnant women enrolled and retained in the final analysis of the BUNMAP cohort, Butajira Ethiopia 2018–2019. BUNMAP, Butajira Nutrition, Mental health, and Pregnancy; U/S, ultrasound

In the BUNMAP Project, a total of 1,860 women were screened and 1,024 of them met the inclusion criteria. Out of the 1,024 women who were enrolled in the study, 196 women exited from the study. Eighty-two women (17 twin pregnancies, four fetuses with congenital anomalies, eight abortions, and 53 women with only single ultrasound measurement in the gestational age 14–38 weeks) were excluded from the analysis (Fig. 1).

We analyzed a total of 2,055 ultrasound scans among 746 singleton fetuses. In the present analysis, we included pregnant women with 2 and above ultrasound measurements from 14–38 weeks of gestation. Growth patterns were analyzed, and growth charts were developed based on the estimated fetal weight (EFW) of 746 singleton fetuses. Percentiles of estimated fetal weight and biometric measurements were analyzed for fetuses above 14 weeks of gestation. Percentiles for small for gestational age was analyzed among fetuses above 22 weeks of gestation.

### Baseline maternal characteristics

The median age of the women during enrolment was 26 years (interquartile range, 23–30). Almost all (745/746) of them were married, 85.5% were Muslim by religion and 71.3% are Guragie by ethnicity. Majority of them (75.6%) were housewife, 48% completed their primary education (grade 1–8), and 29.5% were illiterate. The median mid-upper arm circumference was 24.6 cm (interquartile range, 23.4- 26.5), and 23.1% of the women have mid upper arm circumference below 23 cm. The detail socio demographic characteristics of participants shown in Table 1.

**Table 1:** Socio-demographic characteristics of mothers participated in the study, Butajira Ethiopia, 2018–2019

**Age**	
15–24	232 (31.1)
25–34	467 (62.6)
35–44	47 (6.3)
**Educational status**	
Completed College/ University	11(1.5)
Completed secondary education	75 (10.1)
Primary (grade 1–8)	358 (48)
Read &write	82 (11)
illiterate	220 (29.5)
**Ethnicity**	
Guragie	532 (71.3)
Siltie	161 (21.6)
Other	53 (7.1)
**Religion**	
Muslim	638 (85.5)
Orthodox	74 (9.9)
protestant	34 (4.6)
**Occupation**	
Housewife	564 (75.6)
Farmer	70 (9.4)
Merchant	66 (8.8)
Other	46 (6.2)
**Residency**	
Urban	149 (20)
Rural	597(80)
**Hemoglobin (*n* = 731)**	
< 11gm/dl	61 (8.2)
≥ 11gm/dl	670 (89.8)

### Intrauterine growth evaluation

The median number of ultrasound scan per fetus was 3 ranging from 2–6. Nearly, 48% (357/746) had three ultrasound scans, 37.3% (278/746) had two ultrasound scans, and 13.4% (100/746) had four ultrasound scans between 14–38 weeks. Out of the 2,055 ultrasound scans, 52.7% (1084/2055) scans done in the gestational age 25–35 weeks, 33.8% (694/2055) in 14–24 weeks and 13.5% (277/2055) done in 36–38 weeks of gestation. The distribution of ultrasound examination and descriptive statistics of EFW by gestational age presented in Table 2 (placed at the end of the manuscript). It shows that the coefficient of variation decreased as the gestational age increased.

**Table 2:** Descriptive characteristics of estimated fetal weight and distribution of ultrasound examinations in relation to gestational age, Butajira Ethiopia, 2018–2019

**Gestational age (weeks**)	**Number of observations**	**Estimated fetal weight (g)**			
		Minimum	Maximum	Mean ± SD	CV%
**14**	60	68	107	83 ± 9	10.8
**15**	52	90	127	111 ± 9	8.1
**16**	59	108	177	140 ± 13	9.3
**17**	65	143	209	173 ± 14	8.1
**18**	59	183	290	215 ± 22	10.2
**19**	53	200	328	271 ± 25	9.2
**20**	64	199	416	320 ± 31	9.7
**21**	80	232	486	383 ± 35	9.1
**22**	72	387	544	466 ± 35	7.5
**23**	67	473	780	553 ± 50	9
**24**	63	500	749	646 ± 46	7.1
**25**	93	593	980	756 ± 58	7.7
**26**	102	615	1036	845 ± 66	7.8
**27**	137	798	1408	974 ± 74	7.6
**28**	104	940	1315	2000 ± 78	3.9
**29**	76	993	1559	1265 ± 91	7.2
**30**	78	1171	1636	1415 ± 99	7
**31**	94	1194	1860	1572 ± 124	7.9
**32**	58	1439	2043	1780 ± 119	6.7
**33**	86	1528	2325	2008 ± 145	7.2
**34**	126	1800	2521	2208 ± 129	5.8
**35**	130	2042	2999	2446 ± 169	6.9
**36**	134	2234	3091	2708 ± 164	6.1
**37**	94	2558	3554	2957 ± 172	5.8
**38**	49	2825	3515	3173 ± 137	4.3

**Fig. 2:**
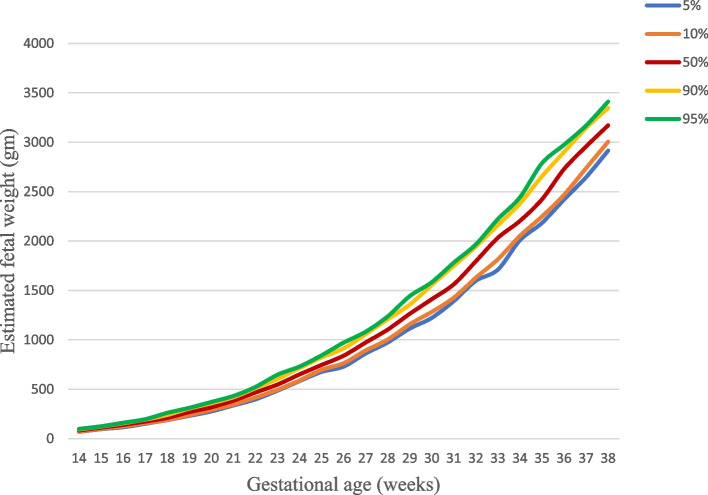
Estimated fetal weight (gm) by percentiles (14–38 weeks), Butajira Ethiopia 2018–2019. Distribution of the 5^th^,10^th^,50^th^, 90^th^, and 95^th^ percentiles of estimated fetal weight, *n* = 2055 observations

**Fig. 3:**
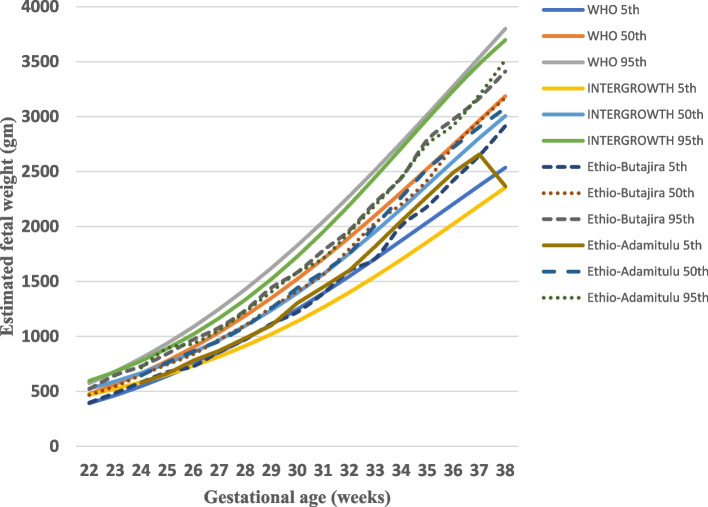
Distribution of the 5^th^, 50^th^ and 95^th^ percentiles for estimated fetal weight for gestational ages 22 to 38 weeks. Selected percentiles taken from WHO study [6], INTERGROWTH-21^st^ study [8], Butajira-Ethiopia (current study) and Adamitulu-Ethiopia study [10]

We plotted smoothed 5^th^,10^th^, 50^th^, 90^th^ and 95^th^ percentiles of estimated fetal weight as shown in Fig. 2. The growth chart shows similar growth pattern with the standards. The growth pattern distribution in comparison to different studies (Ethio-Adamitulu, WHO and INTERGROWTH studies) is also presented in Fig. 3.

**Fig. 4:**
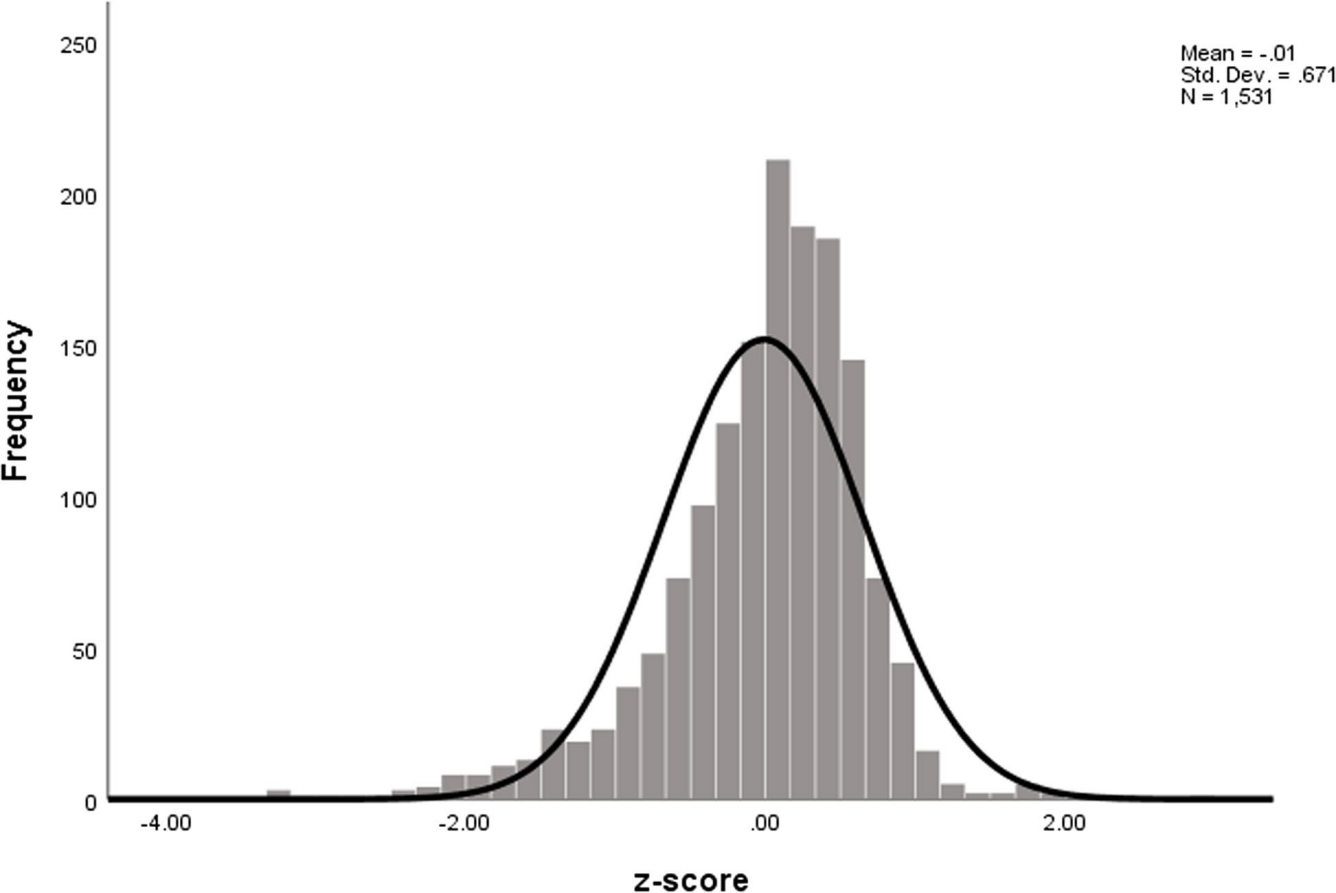
Z-score of estimated fetal weight, Butajira Ethiopia, 2018–2019. The Z-score distribution ranged from -3.33 to 2.99. *n* = 1,531 observations for the gestational age ≥ 22 weeks included in the analysis

To calculate the z-score for EFW, we included a total of 1,531 ultrasound scans whose gestational age is ≥ 22 weeks. The z-score for estimated fetal weight ranged from -3.33 to 2.99, with a mean of -0.01 ± 0.67 as shown in Fig. 4. We found that 10% of the fetus were small for gestational age (below the 10^th^ percentile) based on the Z-score of EFW. Small for gestational age was also found to be 10.1% based on the Z- score of abdominal circumferences.

**Fig. 5:**
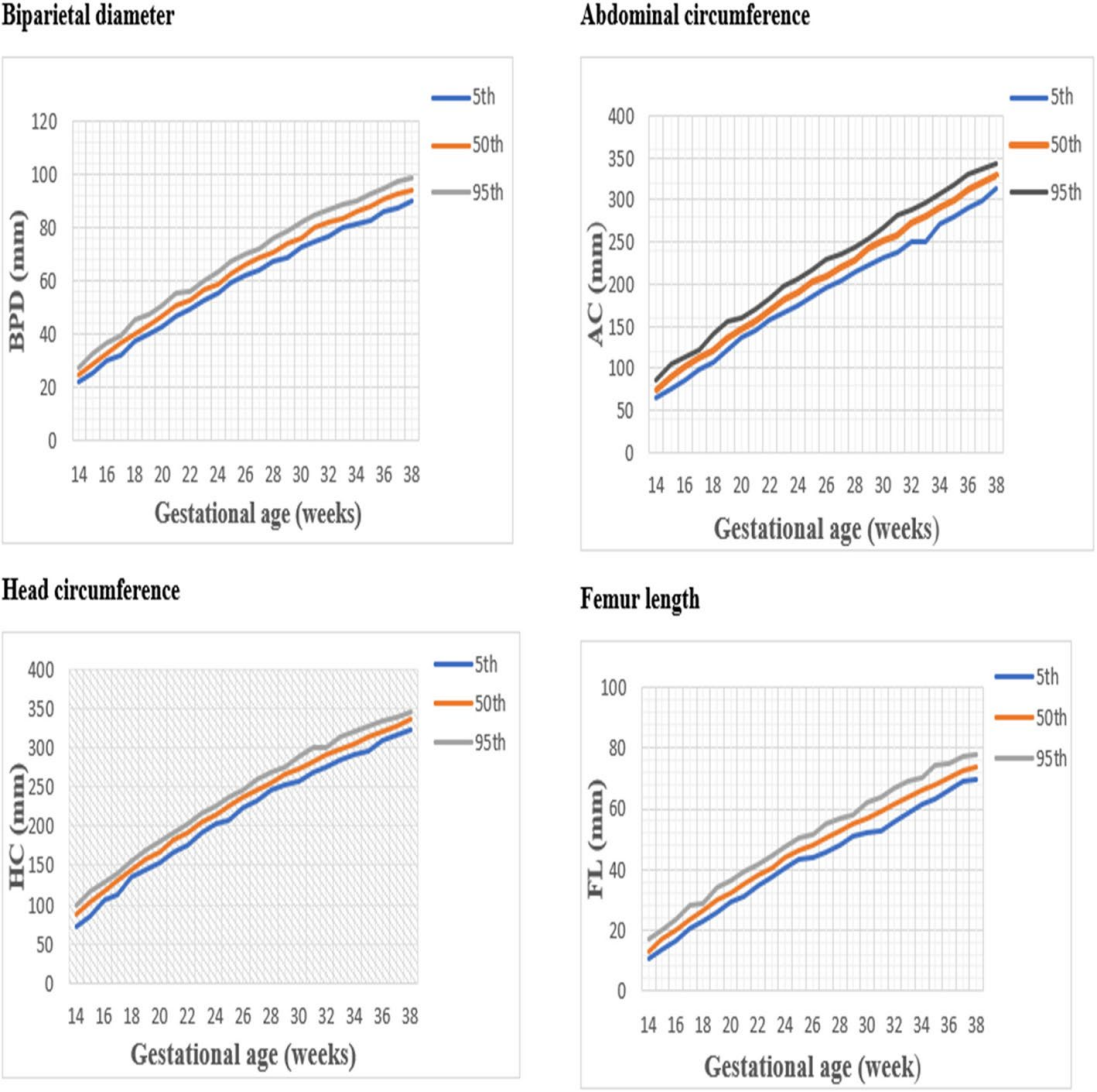
Fetal anthropometric measurements by percentiles and gestational age, Butajira Ethiopia, 2018–2019. Distributions of the 5^th^, 50^th^ and 95^th^ percentiles of fetal biometrics, *n* = 2,055 observations for the gestational age 14–38 weeks. BPD, Biparietal diameter; HC, Head circumference; AC, Abdominal circumference; FL, Femur length

The 5^th^,50^th^ and 95^th^ percentiles of each biometric measurements presented in Fig. 5. It shows that each biometrics percentiles for GA range between 14 and 38 weeks at weekly intervals. The growth patterns of each biometrics (AC, BPD, FL, HC) by percentiles presented in S Table 1, S Table 2, S Table 3, and S Table 4 respectively. These growth pattern can be used for charting the four biometric values at a particular GA and this will help for the identification of pregnancies affected by intrauterine growth restriction and macrocosmic babies.

We presented the distribution of 10^th^ and 90^th^ percentiles of the EFW in relation to other studies in Table 3.

**Table 3:** The 10^th^ and 90^th^ percentile for estimated fetal weight in relation to other reference values, Butajira Ethiopia, 2018–2019

Reference Charts	Gestational week				
	**20**	**24**	**28**	**32**	**36**
**10**^**th**^** percentile of EFW (g)**					
INTERGROWTH-21^st^		602	951	1472	2146
**Butajira (Ethiopia)**	**292**	**588**	**1003**	**1630**	**2468**
Adamitulu (Ethiopia)		599	1016	1614	2550
WHO	286	576	1026	1635	2352
**90**^**th**^** percentile of EFW (g)**					
Adamitulu (Ethiopia)		710	1194	1892	2869
**Butajira (Ethiopia)**	**359**	**714**	**1208**	**1943**	**2901**
INTERGROWTH-21^st^		751	1277	2090	3086
WHO	380	765	1368	2187	3153

Percentiles from previous study in Ethiopia [10], the present study (bold), INTERGROWTH-21^st^ study [8], and WHO study [6], are listed according to ascending values at 28 weeks but are not formally compared or ranked

*AC* Abdominal circumference, *EFW* Estimated fetal weight

As shown in S Table 5, S Table 6, and S Table 7, we calculated the FL/HC, FL/BPD and FL/AC ratios to assess fetal asymmetry. Our finding shows that 2.6% (54/2055 US scans) had microcephaly (BPD < 3 SD). In this study, we also found that the mean FL/HC, FL/BPD and FL/AC ratios were 20 ± 2%,73 ± 8% and 22 ± 2% respectively.

## Discussion

We described the intrauterine growth pattern in rural community of Ethiopia. We found that the intrauterine growth pattern was similar to the previous studies. The study found that 10% of the fetus were small for gestational age (below the 10^th^ percentile) based on the Z-score of EFW. We also found that 10.1% of the fetus were small for gestational age based on the Z- score of abdominal circumferences.

Our study shows similar distribution pattern with the international reference standards, However, at 37 weeks of gestation, we found the 5^th^,50^th^ and 95^th^ percentiles of EFW 2,646, 2,958 and 3,168 gm while the WHO and INTERGROWTH-21^st^ studies found 2,372, 2,966, 3,538 gm and 2,190, 2,806, 3,480 gm respectively. When we compared with WHO and INTERGROWTH studies, the 95^th^ percentiles of EFW in this study is lower than them. The difference might be due to the sample size differences between our study and the WHO and INTERGROWTH studies. Participant difference might also bring such differences; INTERGROWTH-21^st^ and WHO studies took well-nourished participants without known health, environmental, and/or socioeconomic constraints. Besides, living at an altitude lower than 1,500 m was also their inclusion criteria while our study area has an average altitude of 2,500 m above sea level with a range of 1,500 m in the low lands to 3,500 m in the mountainous areas [11]. This high altitude might cause hypoxia which is an important factor to affect fetal development during pregnancy due to limited oxygen to meet the needs of fetal growth and development [21].

The Z- score distributions of BPD, HC, AC and FL in our study ranges from -4.9 to + 2.8, - 4.7 to + 4.1, -5.7 to + 3.7 and -6.1 to + 6.9 respectively. When we fit to the INTERGROWTH-21^st^ application software, the Z-score distribution of biometric measurements in our study were different and lower than the INTERGROWTH 21^st^ standards which is between -3 & + 3. For example, z-score distribution of BPD in our study is between -4.9 and + 2.8. This variation might be due to the measurement difference; in our study BPD was measured from outer to inner diameter of the parietal bone, while INTERGROWTH-21^st^ measures from outer-to-outer diameter [8]. On average, an estimated increase of 0.06 mm in difference between outer-to-outer and outer-to-inner measurements for every one-week increase in gestational age was observed from the previous study [22]. Other possible reasons for lower Z-score distributions of biometrics in our study might be due to study participant characteristics. The INTERGROWTH-21^st^ study includes those with adequate nutritional status [23]. This resulted in a group of clinically healthy women, with adequate nutritional status, who by definition were at low risk of fetal growth restriction [8].

The rate of SGA in this study is consistent with the previous study done in the drought affected rural parts of Ethiopia and Tanzania [10, 24]. This similarity between our study and the previous study conducted in the drought affected areas of Ethiopia where more than 40% of the study participants were undernourished at the start of the study, might be explained by placental adaptation; maternal undernutrition is associated with degenerative placenta changes. However, degenerative placental changes may not impair the placenta’s transport capacity sufficiently to alter fetal nutrition and were not thereby associated with reduction in fetal growth [25].

In our study, both EFW and AC z-scores below the 10^th^ percentile gave similar rate of SGA. This indicates, AC and EFW below the 10^th^ percentile can be used to diagnose small for gestational age. Different research also examined the ability of AC in detecting SGA and fetal growth restriction. They found that AC below the 10^th^ percentile can be used to diagnose FGR [26, 27].

We calculated the fetal biometrics ratios to see the fetal body proportion. A literature says that if the 90^th^ percentile of the FL/AC ratio ˃23.5%, the fetus is growth retarded [28]. In our study the 90^th^ percentile is above the recommended values which is 24.4% and suggestive of fetal growth retardation. Hohler and Quetel also stated that a FL/BPD ratio of 79 ± 8% after 22 weeks of gestation is normal. Values above the specified number considered as microcephaly and lesser values considered as short limbed dwarfism [29]. In our case we found that 75.3 ± 5.5% FL/BPD ratio which can be suggestive of short limbed dwarfism. When we compared the FL/HC ratio with the WHO fetal growth charts, our finding is much lower than the WHO standards.

### Strength and limitation

The current study had many strengths. We have used longitudinal study that gave reference intervals for both fetal size and growth pattern unlike cross-sectional studies that provide information on fetal size only. This study included the relatively large number of study participants from both urban and rural settings which can give representative research findings. The study is part of an on ongoing project under the school of public health in Addis Ababa University in that link the current study findings with subsequent growth and developmental studies. Moreover, studying in a HDSS site has its own value; study participants are under the follow up of the HDSS and will be used for further studies too. Another strength of the study is the use of ultrasound to estimate GA and EFW, which is the gold standard for the assessment of fetal growth. This will give a robust finding and represent all fetus in full second and third trimester.

The present study had some limitations. Participants in this study were not coming at a regular interval of time for ultrasound examination. This result in difficulties to identify in which trimester the growth faltering was more common. In addition, this study might not show whether growth faltering occurs in later gestational ages (after 38 weeks).

## Conclusion and recommendations

Our study evaluated the intrauterine growth patten using ultrasound measurements. We found that the fetal growth pattern in our study is similar with the previous study conducted in drought affected areas of Ethiopia, the WHO and INTERGROWTH studies. Our study also found that 10% of the fetus were small for their gestational age. Therefore, we recommend subsequent follow up studies to identify the causes of growth restriction and small fetal size in the study area to confirm whether the small fetal size is due to other causes or the nature of the population in the study area.

### Supplementary Information

**Additional file 1: S Table 1.** Growth chart for fetal abdominal circumference, Butajira Ethiopia, 2018-2019.

**Additional file 2: S Table 2.** Growth chart for fetal biparietal diameter (outer-inner) Butajira Ethiopia, 2018-2019.

**Additional file 3: S Table 3.** Growth charts for fetal femur length Butajira Ethiopia, 2018-2019.

**Additional file 4: S Table 4.** Growth chart for fetal head circumference Butajira Ethiopia, 2018-2019.

**Additional file 5: S Table 5.** Growth chart for fetal femur length/head circumference ratio, Butajira- Ethiopia, 2018-2019.

**Additional file 6: S Table 6.** Growth chart for fetal femur length/biparietal diameter, Butajira Ethiopia, 2018-2019.

**Additional file 7: S Table 7.** Growth chart for fetal femur length/Abdominal circumference ratio, Butajira Ethiopia,2018-2019.

## Data Availability

Data sets used and/or analyzed during the current study is available from the corresponding author on reasonable request.

## Abbreviations

AC: Abdominal Circumference
BMI: Body Mass Index
BPD: Biparietal Diameter
BUNMAP: Butajira, Nutrition, Mental health and Pregnancy
CRL: Crown-rump Length
EFW: Estimated Fetal Weight
FL: Femur Length
GA: Gestational Age
HC: Head Circumference
HDSS: Health and Demographic Surveillance System (HDSS)
IQR: Interquartile Range
IUG: Intra-uterine Growth
MUAC: Mid Upper Arm Circumference
WHO: World Health Organization
