# Supplementary material for: Intrauterine growth pattern in Butajira HDSS, Southern Ethiopia: BUNMAP pregnancy cohort
Source: BMC Pediatr. 2023 Aug 24;23:422. doi: 10.1186/s12887-023-04244-2 (PMC10464298; doi:10.1186/s12887-023-04244-2)
Supplement: Supplementary file 1 — Additional file 1: S Table 1. Growth chart for fetal abdominal circumference, Butajira Ethiopia, 2018-2019. [file 12887_2023_4244_MOESM1_ESM.docx]

| GA (weeks) | Abdominal circumference (mm) by percentiles | | | | | | |
| --- | --- | --- | --- | --- | --- | --- | --- |
|  | **5^th^** | **10^th^** | **25^th^** | **50^th^** | **75^th^** | **90^th^** | **95^th^** |
| 14 | 64.3 | 65.5 | 69.9 | 73.8 | 78.9 | 82.6 | 85.8 |
| 15 | 75.6 | 79.4 | 84.9 | 89.7 | 93.7 | 98.9 | 104.8 |
| 16 | 85.4 | 93.9 | 96.6 | 102.0 | 107.0 | 109.0 | 113.0 |
| 17 | 99.1 | 102.6 | 107.5 | 112.0 | 117.0 | 120.4 | 122.7 |
| 18 | 108.0 | 113.0 | 117.0 | 121.0 | 130.0 | 135.0 | 140.0 |
| 19 | 121.7 | 124.4 | 130.5 | 136.0 | 142.5 | 149.0 | 156.3 |
| 20 | 136.5 | 139.0 | 142.3 | 147.0 | 151.8 | 155.5 | 159.8 |
| 21 | 144.1 | 146.1 | 151.0 | 157.0 | 162.8 | 167.9 | 170.9 |
| 22 | 158.7 | 160.0 | 164.0 | 169.0 | 177.0 | 182.0 | 183.7 |
| 23 | 165.4 | 168.8 | 173.0 | 182.0 | 189.0 | 194.4 | 198.2 |
| 24 | 173.6 | 181.4 | 185.0 | 191.0 | 197.0 | 203.0 | 205.6 |
| 25 | 185.7 | 192.0 | 196.0 | 202.0 | 207.5 | 212.0 | 216.5 |
| 26 | 195.0 | 199.0 | 203.0 | 209.5 | 217.0 | 219.0 | 229.0 |
| 27 | 205.0 | 207.8 | 214.0 | 220.0 | 226.0 | 231.2 | 235.1 |
| 28 | 214.5 | 218.0 | 223.3 | 228.0 | 236.0 | 241.5 | 244.0 |
| 29 | 223.0 | 230.0 | 236.0 | 242.0 | 246.0 | 252.0 | 255.0 |
| 30 | 229.7 | 236.5 | 244.8 | 251.0 | 257.0 | 263.2 | 267.2 |
| 31 | 236.8 | 241.5 | 249.0 | 258.5 | 266.3 | 273.5 | 281.5 |
| 32 | 250.7 | 254.0 | 262.8 | 272.0 | 278.0 | 284.2 | 288.1 |
| 33 | 250.1 | 264.7 | 276.0 | 282.0 | 289.3 | 295.3 | 297.7 |
| 34 | 272.4 | 277.7 | 285.0 | 291.0 | 298.3 | 306.0 | 308.0 |
| 35 | 279.0 | 285.0 | 292.0 | 301.0 | 309.0 | 314.0 | 317.9 |
| 36 | 289.8 | 297.0 | 305.0 | 313.0 | 320.0 | 324.0 | 330.0 |
| 37 | 298.8 | 309.0 | 316.0 | 321.5 | 328.3 | 334.0 | 336.5 |
| 38 | 313.0 | 319.0 | 321.5 | 330.0 | 334.0 | 340.0 | 342.5 |

**S Table 1: Growth chart for fetal abdominal circumference, Butajira Ethiopia, 2018-2019.**
