# Supplementary material for: Intrauterine growth pattern in Butajira HDSS, Southern Ethiopia: BUNMAP pregnancy cohort
Source: BMC Pediatr. 2023 Aug 24;23:422. doi: 10.1186/s12887-023-04244-2 (PMC10464298; doi:10.1186/s12887-023-04244-2)
Supplement: Supplementary file 2 — Additional file 2: S Table 2. Growth chart for fetal biparietal diameter (outer-inner) Butajira Ethiopia, 2018-2019. [file 12887_2023_4244_MOESM2_ESM.docx]

| GA (weeks) | Biparietal diameter (mm) by percentiles | | | | | | |
| --- | --- | --- | --- | --- | --- | --- | --- |
|  | **5^th^** | **10^th^** | **25^th^** | **50^th^** | **75^th^** | **90^th^** | **95^th^** |
| 14 | 21.6 | 22.1 | 23.1 | 24.7 | 26.1 | 26.9 | 27.2 |
| 15 | 25.4 | 26.6 | 27.5 | 28.6 | 30.0 | 31.4 | 32.5 |
| 16 | 29.9 | 30.1 | 31.6 | 32.8 | 34.3 | 36.1 | 36.8 |
| 17 | 31.8 | 33.9 | 35.5 | 36.9 | 38.0 | 39.1 | 39.5 |
| 18 | 37.0 | 37.3 | 39.0 | 39.9 | 41.4 | 42.4 | 45.0 |
| 19 | 39.9 | 40.4 | 41.7 | 43.5 | 44.9 | 45.8 | 47.4 |
| 20 | 42.5 | 44.4 | 45.3 | 46.4 | 48.1 | 49.1 | 50.5 |
| 21 | 46.8 | 47.4 | 49.1 | 50.4 | 51.6 | 53.8 | 55.2 |
| 22 | 49.5 | 50.3 | 51.5 | 52.9 | 54.2 | 55.7 | 56.1 |
| 23 | 52.3 | 53.9 | 55.4 | 56.6 | 57.9 | 59.1 | 60.2 |
| 24 | 55.2 | 55.8 | 57.3 | 58.7 | 60.0 | 60.7 | 63.2 |
| 25 | 58.9 | 59.4 | 60.9 | 62.7 | 64.1 | 65.5 | 67.0 |
| 26 | 61.9 | 63.1 | 64.3 | 65.7 | 67.4 | 69.3 | 69.7 |
| 27 | 63.9 | 64.8 | 66.5 | 68.3 | 69.8 | 71.1 | 71.9 |
| 28 | 67.2 | 68.4 | 69.5 | 70.8 | 73.1 | 74.9 | 75.8 |
| 29 | 68.4 | 70.2 | 71.7 | 73.7 | 75.8 | 77.5 | 78.6 |
| 30 | 72.4 | 73.4 | 74.4 | 76.2 | 77.7 | 79.3 | 81.8 |
| 31 | 74.5 | 75.2 | 77.4 | 80.0 | 82.1 | 83.9 | 84.4 |
| 32 | 76.7 | 77.6 | 79.2 | 82.2 | 83.6 | 85.4 | 86.8 |
| 33 | 80.0 | 80.6 | 81.8 | 83.5 | 85.0 | 86.9 | 88.5 |
| 34 | 81.0 | 82.7 | 84.6 | 86.1 | 87.9 | 89.3 | 90.1 |
| 35 | 82.4 | 84.2 | 86.1 | 87.6 | 89.9 | 91.3 | 92.5 |
| 36 | 85.5 | 86.5 | 88.8 | 90.3 | 91.7 | 93.7 | 94.4 |
| 37 | 87.5 | 87.9 | 90.5 | 92.4 | 93.6 | 95.4 | 96.9 |
| 38 | 89.9 | 91.3 | 92.9 | 94.1 | 96.3 | 97.7 | 98.2 |

S Table 2: Growth chart for fetal biparietal diameter (outer-inner) Butajira Ethiopia, 2018-2019.
