# Supplementary material for: Intrauterine growth pattern in Butajira HDSS, Southern Ethiopia: BUNMAP pregnancy cohort
Source: BMC Pediatr. 2023 Aug 24;23:422. doi: 10.1186/s12887-023-04244-2 (PMC10464298; doi:10.1186/s12887-023-04244-2)
Supplement: Supplementary file 3 — Additional file 3: S Table 3. Growth charts for fetal femur length Butajira Ethiopia, 2018-2019. [file 12887_2023_4244_MOESM3_ESM.docx]

| GA (weeks) | Femur length (mm) by percentiles | | | | | | |
| --- | --- | --- | --- | --- | --- | --- | --- |
|  | **5^th^** | **10^th^** | **25^th^** | **50^th^** | **75^th^** | **90^th^** | **95^th^** |
| 14 | 10.4 | 10.9 | 11.8 | 13.2 | 14.2 | 15.4 | 16.9 |
| 15 | 13.7 | 14.2 | 15.5 | 16.8 | 18.0 | 19.4 | 20.2 |
| 16 | 16.4 | 17.1 | 18.2 | 19.7 | 20.8 | 22.3 | 23.3 |
| 17 | 20.3 | 20.5 | 21.8 | 23.3 | 24.4 | 26.5 | 28.4 |
| 18 | 23.0 | 24.0 | 24.9 | 26.3 | 26.9 | 28.4 | 28.9 |
| 19 | 25.8 | 27.0 | 28.5 | 30.0 | 30.8 | 32.2 | 33.8 |
| 20 | 29.4 | 30.0 | 31.3 | 32.3 | 34.0 | 34.9 | 36.1 |
| 21 | 31.1 | 32.4 | 34.2 | 35.0 | 36.2 | 37.2 | 39.0 |
| 22 | 34.3 | 35.6 | 36.6 | 38.2 | 39.8 | 41.1 | 41.6 |
| 23 | 37.4 | 37.9 | 39.4 | 40.4 | 41.6 | 43.2 | 44.4 |
| 24 | 40.6 | 41.4 | 42.5 | 44.0 | 44.8 | 46.5 | 47.4 |
| 25 | 43.1 | 43.8 | 45.2 | 46.3 | 47.6 | 49.3 | 50.2 |
| 26 | 44.2 | 45.1 | 46.1 | 48.0 | 49.9 | 51.0 | 51.5 |
| 27 | 45.7 | 47.5 | 48.7 | 50.4 | 51.7 | 53.3 | 55.1 |
| 28 | 48.1 | 49.5 | 51.0 | 52.7 | 54.1 | 55.5 | 56.7 |
| 29 | 50.7 | 52.0 | 53.1 | 54.8 | 56.2 | 57.4 | 57.9 |
| 30 | 52.3 | 53.2 | 54.8 | 56.6 | 58.3 | 60.7 | 62.2 |
| 31 | 52.8 | 55.1 | 57.0 | 59.2 | 60.9 | 62.9 | 63.9 |
| 32 | 55.9 | 57.8 | 59.6 | 61.6 | 63.3 | 64.8 | 66.5 |
| 33 | 58.8 | 59.4 | 61.7 | 64.1 | 65.9 | 68.2 | 69.0 |
| 34 | 61.5 | 62.1 | 63.8 | 65.9 | 67.3 | 69.1 | 70.0 |
| 35 | 63.2 | 64.2 | 66.0 | 68.1 | 70.3 | 71.8 | 74.2 |
| 36 | 65.9 | 66.8 | 68.8 | 70.3 | 72.0 | 74.0 | 75.0 |
| 37 | 68.8 | 69.4 | 70.9 | 72.3 | 74.1 | 76.4 | 77.0 |
| 38 | 69.6 | 70.6 | 72.4 | 74.0 | 75.6 | 77.0 | 77.9 |

**S Table 3: Growth charts for fetal femur length Butajira Ethiopia, 2018-2019.**
