# Supplementary material for: Intrauterine growth pattern in Butajira HDSS, Southern Ethiopia: BUNMAP pregnancy cohort
Source: BMC Pediatr. 2023 Aug 24;23:422. doi: 10.1186/s12887-023-04244-2 (PMC10464298; doi:10.1186/s12887-023-04244-2)
Supplement: Supplementary file 4 — Additional file 4: S Table 4. Growth chart for fetal head circumference Butajira Ethiopia, 2018-2019. [file 12887_2023_4244_MOESM4_ESM.docx]

| GA (weeks) | Head circumference (mm) by percentiles | | | | | | |
| --- | --- | --- | --- | --- | --- | --- | --- |
|  | **5^th^** | **10^th^** | **25^th^** | **50^th^** | **75^th^** | **90^th^** | **95^th^** |
| 14 | 72.3 | 76.9 | 80.3 | 87.0 | 91.6 | 97.7 | 99.2 |
| 15 | 86.2 | 90.4 | 95.4 | 103.5 | 107.5 | 113.7 | 117.0 |
| 16 | 105.0 | 107.0 | 111.0 | 118.0 | 123.0 | 127.0 | 128.0 |
| 17 | 112.6 | 117.4 | 126.0 | 130.0 | 135.0 | 137.4 | 139.0 |
| 18 | 135.0 | 135.0 | 139.0 | 144.0 | 150.0 | 154.0 | 156.0 |
| 19 | 144.7 | 149.0 | 154.0 | 159.0 | 163.5 | 166.2 | 168.9 |
| 20 | 152.3 | 156.5 | 163.0 | 167.0 | 172.8 | 177.0 | 179.5 |
| 21 | 167.0 | 169.1 | 177.0 | 182.0 | 187.0 | 190.9 | 192.0 |
| 22 | 176.7 | 179.0 | 186.0 | 192.0 | 197.0 | 200.0 | 203.1 |
| 23 | 191.4 | 195.8 | 201.0 | 206.0 | 210.0 | 215.0 | 216.6 |
| 24 | 202.0 | 204.0 | 209.0 | 215.0 | 221.0 | 223.0 | 225.6 |
| 25 | 206.7 | 210.2 | 219.0 | 225.0 | 230.0 | 235.6 | 238.0 |
| 26 | 223.0 | 225.0 | 232.8 | 238.0 | 242.0 | 246.0 | 247.0 |
| 27 | 231.9 | 234.8 | 240.0 | 245.0 | 251.0 | 257.2 | 260.1 |
| 28 | 245.0 | 247.0 | 251.0 | 255.0 | 260.0 | 265.0 | 267.8 |
| 29 | 253.9 | 255.7 | 262.0 | 266.0 | 268.8 | 272.3 | 275.0 |
| 30 | 258.0 | 261.0 | 268.8 | 273.5 | 280.0 | 284.1 | 290.1 |
| 31 | 268.0 | 271.0 | 277.0 | 283.0 | 287.3 | 293.5 | 300.3 |
| 32 | 275.9 | 279.7 | 285.0 | 290.5 | 296.3 | 299.2 | 301.3 |
| 33 | 284.0 | 288.7 | 292.0 | 298.0 | 304.0 | 311.0 | 314.7 |
| 34 | 292.0 | 295.0 | 301.0 | 306.0 | 312.0 | 317.3 | 320.7 |
| 35 | 296.6 | 302.1 | 309.0 | 314.0 | 321.0 | 325.0 | 327.9 |
| 36 | 310.0 | 311.5 | 316.0 | 321.0 | 327.0 | 331.5 | 334.5 |
| 37 | 316.8 | 318.5 | 322.0 | 327.0 | 331.0 | 335.5 | 339.0 |
| 38 | 323.0 | 328.0 | 330.0 | 336.0 | 341.0 | 345.0 | 346.5 |

S Table 4: Growth chart for fetal head circumference Butajira Ethiopia, 2018-2019.
