# Supplementary material for: Intrauterine growth pattern in Butajira HDSS, Southern Ethiopia: BUNMAP pregnancy cohort
Source: BMC Pediatr. 2023 Aug 24;23:422. doi: 10.1186/s12887-023-04244-2 (PMC10464298; doi:10.1186/s12887-023-04244-2)
Supplement: Supplementary file 6 — Additional file 6: S Table 6. Growth chart for fetal femur length/biparietal diameter, Butajira Ethiopia, 2018-2019. [file 12887_2023_4244_MOESM6_ESM.docx]

| GA | Femur length/ biparietal diameter ratio by Percentiles | | | | | | |
| --- | --- | --- | --- | --- | --- | --- | --- |
|  | **5^th^** | **10^th^** | **25^th^** | **50^th^** | **75^th^** | **90^th^** | **95^th^** |
| 14 | 0.42 | 0.43 | 0.48 | 0.53 | 0.58 | 0.63 | 0.65 |
| 15 | 0.47 | 0.50 | 0.52 | 0.58 | 0.64 | 0.67 | 0.76 |
| 16 | 0.48 | 0.52 | 0.56 | 0.60 | 0.64 | 0.69 | 0.71 |
| 17 | 0.53 | 0.55 | 0.59 | 0.64 | 0.67 | 0.74 | 0.84 |
| 18 | 0.56 | 0.58 | 0.62 | 0.64 | 0.68 | 0.73 | 0.75 |
| 19 | 0.60 | 0.61 | 0.65 | 0.68 | 0.72 | 0.76 | 0.83 |
| 20 | 0.63 | 0.64 | 0.66 | 0.69 | 0.72 | 0.76 | 0.82 |
| 21 | 0.61 | 0.63 | 0.66 | 0.69 | 0.73 | 0.76 | 0.79 |
| 22 | 0.62 | 0.67 | 0.69 | 0.72 | 0.75 | 0.80 | 0.82 |
| 23 | 0.64 | 0.66 | 0.69 | 0.71 | 0.74 | 0.77 | 0.84 |
| 24 | 0.67 | 0.69 | 0.72 | 0.75 | 0.77 | 0.80 | 0.83 |
| 25 | 0.68 | 0.69 | 0.71 | 0.74 | 0.77 | 0.82 | 0.84 |
| 26 | 0.65 | 0.66 | 0.69 | 0.73 | 0.77 | 0.80 | 0.81 |
| 27 | 0.66 | 0.68 | 0.70 | 0.74 | 0.77 | 0.81 | 0.83 |
| 28 | 0.66 | 0.67 | 0.71 | 0.74 | 0.77 | 0.80 | 0.82 |
| 29 | 0.65 | 0.68 | 0.71 | 0.74 | 0.77 | 0.82 | 0.84 |
| 30 | 0.66 | 0.68 | 0.72 | 0.73 | 0.77 | 0.82 | 0.83 |
| 31 | 0.64 | 0.67 | 0.70 | 0.74 | 0.77 | 0.83 | 0.86 |
| 32 | 0.67 | 0.68 | 0.73 | 0.76 | 0.79 | 0.83 | 0.83 |
| 33 | 0.67 | 0.70 | 0.74 | 0.77 | 0.80 | 0.83 | 0.84 |
| 34 | 0.70 | 0.72 | 0.73 | 0.76 | 0.79 | 0.82 | 0.84 |
| 35 | 0.69 | 0.71 | 0.74 | 0.77 | 0.81 | 0.84 | 0.88 |
| 36 | 0.71 | 0.72 | 0.75 | 0.78 | 0.81 | 0.84 | 0.86 |
| 37 | 0.72 | 0.74 | 0.76 | 0.79 | 0.82 | 0.85 | 0.87 |
| 38 | 0.72 | 0.73 | 0.75 | 0.79 | 0.81 | 0.84 | 0.85 |

S Table 6: Growth chart for fetal femur length/biparietal diameter, Butajira Ethiopia, 2018-2019.
