# Supplementary material for: Intrauterine growth pattern in Butajira HDSS, Southern Ethiopia: BUNMAP pregnancy cohort
Source: BMC Pediatr. 2023 Aug 24;23:422. doi: 10.1186/s12887-023-04244-2 (PMC10464298; doi:10.1186/s12887-023-04244-2)
Supplement: Supplementary file 7 — Additional file 7: S Table 7. Growth chart for fetal femur length/Abdominal circumference ratio, Butajira Ethiopia,2018-2019. [file 12887_2023_4244_MOESM7_ESM.docx]

| GA | Femur length/ abdominal circumference ratio by Percentiles | | | | | | |
| --- | --- | --- | --- | --- | --- | --- | --- |
|  |  | | | | | | |
|  | **5^th^** | **10^th^** | **25^th^** | **50^th^** | **75^th^** | **90^th^** | **95^th^** |
| 14 | 0.14 | 0.15 | 0.16 | 0.18 | 0.19 | 0.21 | 0.24 |
| 15 | 0.14 | 0.16 | 0.17 | 0.19 | 0.21 | 0.23 | 0.24 |
| 16 | 0.16 | 0.17 | 0.18 | 0.19 | 0.21 | 0.22 | 0.24 |
| 17 | 0.17 | 0.18 | 0.19 | 0.21 | 0.22 | 0.24 | 0.27 |
| 18 | 0.18 | 0.19 | 0.20 | 0.21 | 0.23 | 0.24 | 0.25 |
| 19 | 0.19 | 0.20 | 0.21 | 0.22 | 0.23 | 0.25 | 0.27 |
| 20 | 0.20 | 0.20 | 0.21 | 0.22 | 0.23 | 0.24 | 0.25 |
| 21 | 0.19 | 0.20 | 0.21 | 0.22 | 0.24 | 0.25 | 0.26 |
| 22 | 0.20 | 0.20 | 0.21 | 0.22 | 0.24 | 0.25 | 0.26 |
| 23 | 0.20 | 0.20 | 0.21 | 0.22 | 0.23 | 0.24 | 0.26 |
| 24 | 0.20 | 0.21 | 0.22 | 0.23 | 0.24 | 0.25 | 0.26 |
| 25 | 0.20 | 0.21 | 0.22 | 0.23 | 0.24 | 0.25 | 0.26 |
| 26 | 0.21 | 0.21 | 0.22 | 0.23 | 0.24 | 0.25 | 0.25 |
| 27 | 0.20 | 0.21 | 0.22 | 0.23 | 0.24 | 0.25 | 0.26 |
| 28 | 0.20 | 0.21 | 0.22 | 0.23 | 0.24 | 0.25 | 0.26 |
| 29 | 0.21 | 0.21 | 0.22 | 0.23 | 0.23 | 0.24 | 0.25 |
| 30 | 0.20 | 0.21 | 0.22 | 0.22 | 0.24 | 0.25 | 0.26 |
| 31 | 0.20 | 0.21 | 0.22 | 0.23 | 0.24 | 0.25 | 0.26 |
| 32 | 0.20 | 0.21 | 0.22 | 0.23 | 0.24 | 0.25 | 0.25 |
| 33 | 0.21 | 0.21 | 0.22 | 0.23 | 0.23 | 0.25 | 0.26 |
| 34 | 0.21 | 0.21 | 0.22 | 0.22 | 0.23 | 0.24 | 0.25 |
| 35 | 0.21 | 0.21 | 0.22 | 0.23 | 0.24 | 0.25 | 0.26 |
| 36 | 0.20 | 0.21 | 0.22 | 0.23 | 0.23 | 0.24 | 0.25 |
| 37 | 0.21 | 0.21 | 0.22 | 0.23 | 0.23 | 0.24 | 0.25 |
| 38 | 0.21 | 0.21 | 0.22 | 0.23 | 0.23 | 0.24 | 0.24 |

**S Table 7:** Growth chart for fetal femur length/Abdominal circumference ratio, Butajira Ethiopia,2018-2019.
